# Parasitic chytrids sustain zooplankton growth during inedible algal bloom

**DOI:** 10.3389/fmicb.2014.00229

**Published:** 2014-05-23

**Authors:** Serena Rasconi, Boutheina Grami, Nathalie Niquil, Marlène Jobard, Télesphore Sime-Ngando

**Affiliations:** ^1^CNRS UMR 6250, UMRi 7266, LIENSs, Université de La RochelleLa Rochelle, France; ^2^CNRS UMR 6023, LMGE, Clermont UniversitéAubière Cedex, France

**Keywords:** fungal parasites, bloom, stability, inverse modeling, ecological network analysis

## Abstract

This study assesses the quantitative impact of parasitic chytrids on the planktonic food web of two contrasting freshwater lakes during different algal bloom situations. Carbon-based food web models were used to investigate the effects of chytrids during the spring diatom bloom in Lake Pavin (oligo-mesotrophic) and the autumn cyanobacteria bloom in Lake Aydat (eutrophic). Linear inverse modeling was employed to estimate undetermined flows in both lakes. The Monte Carlo Markov chain linear inverse modeling procedure provided estimates of the ranges of model-derived fluxes. Model results confirm recent theories on the impact of parasites on food web function through grazers and recyclers. During blooms of “inedible” algae (unexploited by planktonic herbivores), the epidemic growth of chytrids channeled 19–20% of the primary production in both lakes through the production of grazer exploitable zoospores. The parasitic throughput represented 50% and 57% of the zooplankton diet, respectively, in the oligo-mesotrophic and in the eutrophic lakes. Parasites also affected ecological network properties such as longer carbon path lengths and loop strength, and contributed to increase the stability of the aquatic food web, notably in the oligo-mesotrophic Lake Pavin.

## Introduction

Parasites are known to be ubiquitous in their environments. Although they have been considered as important forcing factors for ecological processes (Hudson et al., 2006), they have only recently been included in food web studies. In aquatic ecosystems, freshwater parasites are especially common in the form of “zoosporic” fungi (i.e., chytrids). The life cycle of these parasites is characterized by dispersal forms, uniflagellated zoospores, and sporangia attached to the host cells. Microscopic observations provided evidence for the presence of both forms in freshwater ecosystems (Rasconi et al., 2009; Jobard et al., 2010). These parasites mostly affect primary producers (Canter, 1950; Sparrow, 1960), food web dynamics (Mccallum et al., 2004; Sime-Ngando, 2012) and ecological processes (Hudson et al., 2006). Many phytoplankton species are sensitive to chytrid parasites and the related ecological implications are important (Canter and Lund, 1948; Niquil et al., 2011; Sime-Ngando, 2012). Chytrid infections have been linked to mass mortalities of host organisms, to suppression or postponement of phytoplankton blooms, and have selective effects on host species composition and successions (Van Donk and Ringelberg, 1983; Bruning et al., 1992; Kagami et al., 2007; Rasconi et al., 2009). Unstable ecosystems seem to favor the opportunistic behavior of parasites (Rasconi et al., 2012), where their activity represents an important but as yet overlooked ecological driving force in food web dynamics. During blooms, parasites can optimize their virulence, as the host population is genetically more uniform (Brown et al., 2002) and bloom-forming species exhibit short generation time. Some fungal parasites seem to be most common in large and bloom forming algae that are fairly resistant to grazing by zooplankton (Sommer, 1987; Kagami et al., 2007; Rasconi et al., 2012), including diatoms and cyanobacteria. Recent studies have suggested a role for fungal parasites in destroying large filamentous phytoplankton, which are considered important for seasonal pelagic succession (Rasconi et al., 2012; Gerphagnon et al., 2013). This finding raises the hypothesis that parasites may play important roles during monospecific blooms of inedible algae because they can release dissolved substrates for microbial processes through host destruction, and provide energetic particles as zoospores for higher trophic levels (Kagami et al., 2007; Grami et al., 2011).

Diatoms traditionally form large spring blooms in temperate lakes, providing fuel for planktonic community development at the start of the growing season. However, some of the diatom blooms are not grazed by filter-feeding zooplankton due to their large size, and this biomass is believed to be lost by sinking from the euphotic zone instead of being grazed. Recently there has also been an increasing awareness of food quality as a limiting factor for zooplankton growth (Brett and Muller Navarra, 1997; Sterner and Elser, 2009; Kagami et al., 2011). Laboratory studies suggest that a diatom monodiet lacks or is deficient in some essential component required for copepod egg development and may have a harmful effect on the success of egg hatching. Some authors have also highlighted the toxicity of these algae as food and presented evidence showing that the hatching success of wild copepods feeding on a diatom-dominated bloom is heavily compromised (Miralto et al., 1999). If diatoms have a deleterious effect, high diatom abundance could limit secondary production and affect fish production (Irigoien et al., 2002). However, diatoms have traditionally been regarded as providing the bulk of the food that sustains the planktonic seasonal succession and the food chain to top consumers (Sommer et al., 1986). Additionally, spring diatom proliferations are generally followed by a rapid increase in zooplankton. Diatoms are well known as preferential hosts for chytrid epidemics in the plankton (Ibelings et al., 2004; Kagami et al., 2007) and chytrid zoospores were experimentally demonstrated to be efficiently grazed and be able to sustain *Daphnia* growth in *Asterionella* cultures (Kagami et al., 2007). This implies that during fungal epidemics abundant zoospores may become a food source for some grazers. When fungi infect these large inedible phytoplankton species, they consume nutrients within these cells to produce zoospores, some of which are grazed by zooplankton with important consequences for the recycling of the organic matter in the pelagic food web (Kagami et al., 2007; Grami et al., 2011; Niquil et al., 2011).

Cyanobacteria, the most ancient phytoplankton on the planet, have been an important element for forming the earth's oxygen atmosphere. They frequently form blooms and dominate phytoplankton communities in warm, stratified and nutrient-enriched waters. In lakes, they form the basis of the food chain and function as nitrogen fixers. Proliferation events seem to have increased substantially during recent decades, likely as a result of eutrophication and temperature increase; they may have a large impact on water quality and biological communities. Many genera of cyanobacteria are known to produce a wide variety of toxins and bioactive compounds (Sivonen and Jones, 1999), which are a health risk to both animals and humans. From the perspective of aquatic food webs, cyanobacterial blooms can noticeably decrease the efficiency of the energy transfer from primary producers to primary consumers (Lurling and Roessink, 2006). However, filamentous cyanobacteria are known to be the target of different chytrid species (Canter, 1972). A recent hypothesis has been proposed stating that proliferation events may not always represent trophic bottlenecks, since eukaryotic parasites provide energetic particles as zoospores for higher trophic levels (Rasconi et al., 2012; Gerphagnon et al., 2013). Parasitism helps release dissolved substrates for microbial processes through host destruction.

Despite current evidence that bloom situations are considered deleterious for the ecosystem due to harmful and toxic species, blooms can constitute ecologically important events. They contribute to the natural processes of a lake and in some cases provide important benefits by boosting primary productivity and influence the energetics and population dynamics of consumer organisms. Considering the widespread occurrence of parasites during such situations, their consumption likely represents a far more important trophic link than previously recognized. Eukaryotic parasites as consumers drain energy throughout the food web and provide energetic particles for the grazers by predation on free living stages (Kagami et al., 2007; Grami et al., 2011; Miki et al., 2011). Parasitic activity was estimated to constitute between 36% and 44% of observed trophic links in a marine food web (Lafferty et al., 2006). In this paper we analyzed the role of parasitic chytrids and how they affect organic matter transfer in two different algal proliferation contexts: a spring diatom proliferation in an oligo-mesotrophic deep lake, and a late summer cyanobacterial bloom in a eutrophic shallow lake. The aim was to investigate how parasites drive energy and nutrients from their hosts to zooplankton in these two different situations to corroborate our recent findings that the activity of parasites and grazing on parasitic zoospores might sustain the growth of zooplankton through releasing nutrients bound in inedible algae and thus represent an important alternative carbon pathway in pelagic environments (Kagami et al., 2007; Grami et al., 2011; Miki et al., 2011).

Moreover, since we have demonstrated in a previous work (Grami et al., 2011) that parasites drive an increase in species richness, trophic level, connectance, and trophic chain length of the food web, we wanted also to establish the effects of parasites on the ecosystem properties linked to stability during monospecific algal proliferations. In this context of infections of primary producers, predation on parasites occurs at low trophic levels, which is considered a top down effect that reduce loop weight and increase the strength of links (Neutel et al., 2002). Weak to intermediate strength links are important as they can decrease complex oscillatory food-web dynamics and promote community persistence and stability (Mccann et al., 1998). Moreover, food-web stability is linked to species number and connectance, which is enhanced when species at high trophic levels feed on multiple prey or when species at intermediate trophic levels are fed on by multiple predator species (Gross et al., 2009). Parasites can alter topological properties of the network such as patterns of biodiversity, linkage between density and loop strengths, with implications for changing interactive networks and network stability (Lafferty et al., 2008). The significance of such observations is only beginning to be appreciated; integration of parasites has the potential to alter our understanding of food web structure and theory. The impact that parasites have on food web properties like stability and resilience has been previously overlooked and will need to be measured to ascertain its importance in the food web. From a theoretical perspective, predation on algal parasites helps to unite two emerging concepts in plankton ecology: the energetics of the overlooked parasite-grazer system flow during algal biomass proliferation and the impact of this link on the structural and functional properties of the ecosystem.

To investigate parasite-related flows, ecosystem properties and ecological theories, we applied mathematical tools such as linear inverse models for trophic network representation through carbon flows. For the first time we evaluate and compare the impact of zoosporic parasites (“chytrids,” class Chytridiomycetes, families Rhizophidiaceae and Chytridiaceae) on the functioning of a planktonic ecosystem using field data collected from the euphotic zone of two different lake ecosystems in the Massif Central region of France: the oligo-mesotrophic Lake Pavin and the eutrophic Lake Aydat. We compared carbon flows between the complete food web including parasitic chytrids during spring diatom bloom peak in Lake Pavin (April 2007 from 4th to 18th, PavDiat), with the model representative for the fall cyanobacteria bloom in Lake Aydat (from September 24th to October 10th 2007, AydCyan) and quantified the amount of primary production channeled through the food web. These models were built using the Linear Inverse Modeling procedure (LIM, Vezina and Platt, 1988) recently modified into the LIM-Monte Carlo Markov Chain (LIM-MCMC, Van Den Meersche et al., 2009). This method allows reconstruction of missing flow values and alleviates the problem of under-sampling, using the principle of conservation of mass (Vezina and Platt, 1988). The flows obtained from the models were used for calculations of Ecological Network Analysis indices that characterize the structure and functioning properties of the food web, and help reveal emergent properties (Ulanowicz, 1986, 1997; Ulanowicz et al., 2009).

LIM-MCMC and ecological network analysis were used to reveal overlooked trophic links in two contrasted freshwater ecosystems (Pavin and Aydat Lakes) where parasites of microalgae were integrated during bloom periods. Our objectives were to identify and compare (1) the carbon flows involved in the chytrid-parasitism pathway during monospecific algal proliferations in both lakes, (2) the emergent properties of different planktonic food webs during different algal blooms in which parasites are integrated, and (3) the structural and functional properties of two different ecosystems containing different parasite-host associations.

## Materials and methods

### Study site and sampling

Samples were collected in two freshwater lakes with different trophic status located in the French Massif Central. Lake Pavin (45° 29' 41” N, 002° 53' 12” E) is an oligo-mesotrophic, deep volcanic mountain lake (*Z*_max_ = 92 m), with a permanent anoxic monimolimnion from 60 m depth downwards. This site has a small surface area (44 ha), about equal to the drainage basin area (50 ha) and offers a unique environment with low human influences, and consistent annual seasonal dynamics in the water column (Lefêvre et al., 2007, 2008; Rasconi et al., 2012). Lake Aydat (45° 39' 48” N, 02° 59' 04” E) is a small eutrophic lake (*Z*_max_ = 15 m, surface area = 60 ha). Compared to the surface of the lake, the catchment area (3 × 10^4^ ha) is very large and contains intensive agricultural lands.

Samples were collected fortnightly in a central location of each lake by simple capillarity as described by Sime-Ngando and Hartmann (1991). This method allowed collecting integrated samples (21 L) representative of the euphotic layers (0–20 m for Lake Pavin and 0–5 m for Lake Aydat). Samples from Lake Pavin were collected between 4 and 18 April 2007 (diatom bloom), while those considered for the Lake Aydat were collected between 24 September and 10 October 2007 (cyanobacteria bloom). Samples were pre-filtered on 150 μm pore size nylon filter (except for metazooplankton samples) for the elimination of metazoan zooplankton and taken to the laboratory for immediate analysis.

### Abundance and biomass of planktonic organisms

Sub-samples were processed for identification and quantification of picoplankton, heterotrophic nanoflagellates, phytoplankton, zooplankton and the two life stages of microphytoplankton fungal parasites (Chytridiales). Details on the material and methods are available in Grami et al. (2011).

#### Bacteria

Sample aliquots were fixed with glutaraldehyde before counting of heterotrophic and autotrophic picoplankton by a flow cytometer (BD system). Carbon conversion factors of 0.35 pg C μ m^−3^ and 0.22 pg C μ m^−3^ were used for conversion of biovolumes to carbon biomasses of heterotrophic bacteria (*bac*) (Bjornsen, 1986) and picophytoplankton (*ph1*, 0.2–2 μm) (Mullin et al., 1966; Sondergaard et al., 1991), respectively.

#### Heterotrophic nanoflagellates (hnf, 2–20 μm)

Sub-samples (15 ml) were fixed and handled according to Caron (1983) for quantification of heterotrophic nanoflagellates. Counts were performed using an inverted epifluorescent microscope (Leica DMIRB). Mean cell biovolumes were estimated for each sample by measuring the linear dimension of at least 50 cells and equating shapes to standard geometric forms. Carbon biomass was calculated using a conversion factor of 0.22 pg C μ m^−3^ (Børsheim and Bratbak, 1987).

#### Nano- and microphytoplankton (ph2, 2–20 μm and ph3, 20–150 μm, respectively)

Sub-samples (200 ml) were fixed with alkaline Lugol solution (1% v/v) and cells were counted and identified using the Utermohl method (1931) under an inverted microscope (WILD—M40). Cell biovolumes were estimated by measuring the linear dimension of at least 100 cells and equating shapes to standard geometric forms. The resulting volumes were transformed into organic carbon values using the conversion equation of Menden-Deuer and Lessard (2000) (pgC cell^−1^ = 0.288 × Vol^(0,811)^ for diatoms and pgC cell^−1^ = 0.216 × Vol^(0,939)^ for the other autotrophic genera).

#### Ciliates (mic, 20–150 μm)

Sub-samples (200 ml) were fixed with alkaline Lugol solution (5% v/v) and ciliates were counted and identified using the same method as for microphytoplankton. For carbon biomasses of ciliates, biovolumes were converted into organic carbon using conversion factors of 0.19 pg C μm^−3^ (Putt and Stoecker, 1989).

#### Metazooplankton (mes)

The metazooplankton was collected by filtering raw samples from the euphotic layer (0–20 m) through a 50 μm pore-size mesh. Retained animals were preserved in 4% formalin-sucrose (Prepas, 1978). Identification and counting, after addition of few drops of rose Bengal to improve detection, were conducted under a binocular microscope (Wild M3Z) using Dolfuss chambers (Dussart, 1967). The carbon biomass of each metazoan group was estimated by multiplying the individual carbon contents by the corresponding abundances. For Copepods the dry weight (DW, mg) was calculated as 22.5% of wet weight (Riemann et al., 1990; Gradinger et al., 1999) and C content (mg) was estimated as 40% of DW (Feller and Warwick, 1988). For Cladocera the length (L, mm) of each organism was used to determine its carbon content (C_clad_) as: μ g C ind^−1^ = 5.24 × L - 1.08 (Kankaala and Johansson, 1986). For rotifers, wet weights were converted to dry weight according to Pace and Orcutt (1981) and Mccauley (1984). Dry weights were converted to carbon biomass using carbon: DW ratio of 0.48 (Andersen and Hessen, 1991).

#### Chytrid parasites

Sub-samples were handled for chytrid parasites counting based on a size fraction approach and the use of the fluorochrome calcofluor white (CFW) for diagnosing, staining and counting chitinaceous fungal parasites (i.e., sporangia of chytrids) of microphytoplankton (Rasconi et al., 2009). 20 L of the integrated samples were passed through a 25 μm pore size nylon filter. Large phytoplankton cells in the >25 μm size fraction were collected and fixed with formaldehyde (2% final conc.) before staining and analysis.

Nanoplanktonic cells in the <25 μm size-fraction were concentrated by ultrafiltration and 180 ml of the ultrafiltrate retentate was fixed with formaldehyde (2% final conc.), before staining and analysis. Aliquots (150 μ l) of each fraction were stained by CFW (1% v/v) and drops (10 μ l) of stained samples were mounted between glass slides and cover slips for observation and counting under an inverted epifluorescent microscope (more details are available in Rasconi et al., 2009). Identification of chytrids was based on phenotypic keys known from classical manuals, primarily those in Canter (1950); Canter and Lund (1951), and Sparrow (1960). The prevalence of infection was estimated as the percentage of infection in the host population according to Bush et al. (1997), i.e., Pr (%) = [(Ni/N) × 100], where Ni is the number of infected host cells, and N is the total number of host cells. Carbon biomass of sporangia attached to the host cells (*spg*) was estimated using a conversion factor of 10.7 pg C cell^−1^ (Kagami et al., 2007).

For zoospore (*zsp*) counting, sub-samples were processed using the CARD-FISH method of Not et al. (2002), recently modified by Jobard et al. (2010). The number of zoospores produced by sporangia was considered and carbon biomass of zoospores was estimated using a conversion factor of 10.7 pg C cell^−1^ (Kagami et al., 2007).

### Model construction

Data from the field were used to construct pelagic food web models that quantitatively illustrate carbon pathways in Lake Pavin during diatom spring bloom and Lake Aydat during cyanobacteria autumn bloom, in the presence of chytrids (both sporangia and zoospore stages in the life cycle). Since the unknown flows far outnumbered the known flows, we adopted the LIM-MCMC method (Van Den Meersche et al., 2009), derived from the LIM of Vezina and Platt (1988) to reconstruct trophic flows through the pelagic food web. The approach is based on four steps described with more details in Grami et al. (2011).

#### Compartments and a priori model

The first step consists in constructing a conceptual model including all possible flows between compartments and between compartments and the outside. We represented the pelagic food web and carbon pathways in the mycoloop, we thus did not include compartments for fishes and macrophytes. Living compartments included three phytoplankton compartments, three grazer compartments, one compartment for heterotrophic bacteria and two compartments for fungal parasites of microphytoplankton. We divided the phytoplankton into picophytoplankton (*ph1*: 0.2–2 μm); nanophytoplankton (*ph2*: 2–20 μm; principally Cryptophyta as *Rhodomonas* sp. in both lakes and Chlorophyta as *Ankistrodesmus sp.* and *Ankyra sp*. in Lake Pavin) and microphytoplankton (*ph3*: 20–150 μm; essentially large and filamentous Bacillariophyceae, as *Synedra* sp., *Melosira* sp., and *Asterionella* sp. in Lake Pavin and filamentous cyanobacteria as *Anabaena* sp. and *Oscillatoria* sp. in Lake Aydat). Grazer compartments were the heterotrophic nanoflagellates (*hnf*: 2–20 μm), microzooplankton (*mic*; 20–150 μm: Ciliates and small Rotifera) and mesozooplankton (*mes*; >150 mm; Cladocera, Copepoda and some large Rotifera). Phytoplankton fungal parasites compartments included sporangia attached to the host cells (*spg*) and free zoospores (*zsp*). Non-living compartments were dissolved organic carbon (*doc*) and detritus (*det*).

The food web contained 53 carbon flows for the model of Lake Pavin and 54 for the model of Lake Aydat (a flow was added allowing the microzooplankton to consume detrital material). The sole carbon inputs were gross primary production by each phytoplankton size fraction. Carbon output from the network was driven by respiration of all living compartment and carbon loss by sinking from *ph2, ph3, spg, mic, mes*, and *det* compartments. Mesozooplankton contribution to the carbon output flow considers their consumption by higher trophic level and their production of sinking fecal pellets. All living compartments except fungal parasites contribute to the DOC production that was taken up by bacteria. In addition to *ph2, ph3, mic*, and *mes* contribution to detritus production, we considered the existence of a carbon flow from bacteria and heterotrophic nanoflagellates to detritus.

Attached bacteria were identified on TEP—Transparent Exopolymer Particles, associated with vertical flows of carbon in Lake Pavin during spring (Carrias et al., 1998), and then a flow of bacteria to detritus was considered. The bacteria to detritus flow was calculated using data on bacteria attached to TEP sedimenting in Lake Pavin during spring (Lemarchand et al., 2006). These attached bacteria are known to constitute preferential prey for heterotrophic nanoflagellates (Arnous et al., 2010). Detritus production of sporangia was due to chitinaceous wall dissolution or break-up during zoospore discharge (Sparrow, 1960). Moreover, zoospores were considered as contributing to detritus production by the loss of their flagellum when they found a host to fix on. The carbon flow from microphytoplankton to sporangia represented carbon pumped from diatom cells to sporangia and the carbon flow from sporangia to zoospores was considered as the zoospores produced by sporangia. Grazing relationships were defined by considering size and preferential ingestion of each identified grazer. Heterotrophic flagellates grazed on *bac* and *ph1*, microzooplankton grazed on *bac, ph1, ph2, ph3, hnf, zsp*, and *det* (this latter only in Lake Aydat) and mesozooplankton grazed on *bac, ph1, ph2, ph3, hnf, mic, zsp*, and *det*.

#### Equalities

The second step was setting equations (equalities) to constrain the mass balance of the system and to impose measured flows. The mass balance equations for all compartments are given in the first 11 lines of the Table 1. Some of the estimated flows were measured during previous studies that focused on spring blooms in Lake Pavin and are introduced as additional equations (lines 12–15 of Table 1); these include values for total gross and net primary production (Devaux, 1980; Bettarel et al., 2003), bacterial production (Bettarel et al., 2003) and viral lysis of bacteria (Bettarel et al., 2003) considered as the value of the flux from bacteria to DOC. Some other equalities were introduced for the Aydat model and values of total gross primary production (Aleya et al., 1988), bacterivory by heterotrophic nanoflagellates and microzooplankton (Bettarel et al., 2004) were considered (lines 16–18 of Table 1). Primary production values used for Pavin and Aydat were measured from 14C uptake according to Steemann-Nielsen (1952). Bacterial production was determined by measuring the uptake of tritiated thymidine into bacterial DNA (Petit et al., 1999), after incubating the samples for 45 min. Viral lysis of bacteria was considered as the value of the flux from bacteria to DOC. The fraction of bacterial mortality from viral lysis was related to the calculated frequency of visibly infected cells, calculations are detailed in Bettarel et al. (2003). Details on the method are available in Grami et al. (2011) and cited references there in. Values of protozoan bacterivory were calculated using tracer particles and epifluorescence microscopy (EM) following (Pace and Bailiff, 1987) method modified by Carrias et al. (1996), more details are available in Bettarel et al. (2004).

**Table 1:** **Mass balance (1–11) and linear equations used for inverse analysis**.

**Equation number**	**Process concerned**	**Equations**
**COMMON MASS BALANCE EQUATIONS BETWEEN PAVIN AND AYDAT MODELS**		
1	Mass balance for microphytoplankton	(gpp-ph3) − (ph3-res + ph3-doc + ph3-mic + ph3-mes + ph3-spg+ ph3-det + ph3-los) = 0
2	Mass balance for nanophytoplankton	(gpp-ph2) − (ph2-res + ph2-doc + ph2-mic + ph2-mes + ph2-los + ph2-det) = 0
3	Mass balance for picophytoplankton	(gpp-ph1) − (ph1-res + ph1-doc + ph1-hnf + ph1-mic + ph1-mes+) = 0
4	Mass balance for heterotrophic nanoflagellates	(ph1-hnf + bac-hnf) − (hnf-res + hnf-doc + hnf-mic+ hnf-mes + hnf-det) = 0
5	Mass balance for bacteria	(doc-bac) − (bac-res + bac-doc + bac-hnf + bac-mic + bac-mes + bac-det) = 0
6	Mass balance for microzooplankton	(ph1-mic+ ph2-mic + ph3-mic + bac-mic + hnf-mic + det-mic+ zsp-mic) − (mic-res + mic-doc + mic-mes + mic-det+ mic-los) = 0
7	Mass balance for mesozooplankton	(ph1-mes+ ph2-mes + ph3-mes + bac-mes + hnf-mes + mic-mes +det-mes + zsp-mes) − (mes-res + mes-doc + mes-det + mes-los) = 0
8	Mass balance for sporangia	(ph3-spg) − (spg-res + spg-zsp + spg-det+ spg-los) = 0
9	Mass balance for zoospores	(spg-zsp) − (zsp-res + zsp-mic + zsp-mes + zsp-det) = 0
10	Mass balance for detritus	(ph2-det + ph3-det + hnf-det + mic-det + mes-det + bac-det + spg-det + zsp-det) − (det-doc + det-mic^**^+ det-mes + det-los) = 0
11	Mass balance for dissolved organic carbon	(ph1-doc + ph2-doc + ph3-doc + hnf-doc + mic-doc + mes-doc + det-doc) − (doc-bac) = 0
**PAVIN LINEAR EQUALITIES**		
12	Total gross primary production estimate	gpp-ph1 + gpp-ph2 + gpp-ph3 = 676.25^*^
13	Total net primary production estimate	(gpp-ph1 + gpp-ph2 + gpp-ph3) - (ph1-res + ph2-res + ph3-res) = 459.85^*^
14	Net bacterial production	doc-bac − bac-res = 105.60^*^
15	Viral lysis of bacteria	bac-doc = 9.90^*^
**AYDAT LINEAR EQUALITIES**		
16	Total gross primary production estimate	gpp-ph1 + gpp-ph2 + gpp-ph3 = 2520.00^*^
17	Bacterivory by microzooplankton	Bac-hnf = 109.40^*^
18	Bacterivory by heterotrophic nanoflagellates	Bac-mic = 63.02^*^

Gpp-compartment A: gross primary production of compartment A.

Compartment A-Compartment B (e.g., Bac-hnf) represent the carbon flowing from compartment A to compartment B.

Compartment A-res: respiration of compartment A.

Compartment A-los: carbon loss by sedimentation of compartment A.

Bac, bacteria; Ph1, picophytoplankton; Ph2, nanophytoplankton; Ph3, microphytoplankton; Hnf, heterotrophic nanoflagellate; Mic, microzooplankton; Mes, mesozooplankton; Spg, sporangia; Zsp, zoospores; Det, detritus; Doc, dissolved organic carbon.

* values are in mgC m^−2^ d^−1^.

** det-mic was only considered for Aydat model.

#### Constraints

The third step consisted of imposing ecological limits (maximum and/or minimum) for each unknown flow, which means a linear system of inequalities: 76 inequalities where provided for Lake Pavin and 78 inequalities for Lake Aydat. The latter is presented, explained and referenced in Table 2. Details about these inequalities are given in Grami et al. (2011).

**Table 2:** **Constraints used on different planktonic food web processes**.

**Process**		**Bound**	**Description**	**Equations**	**References**
Gross primary production	ph3	Upper and lower	GPP of ph3 is comprised between 60% and 85% of total GPP	60% GPP < GPP-ph3 < 85% GPP	This study
	ph2	Upper and lower	GPP of ph2 is comprised between 2% and 10% of total GPP	2% GPP < GPP-ph2 < 10% GPP	
	ph1	Upper and lower	GPP of ph1 is comprised between 5% and 20% of total GPP	5% GPP < GPP-ph1 < 20% GPP	
Respiration	ph1 ph2	Upper and lower	ph2 and ph1 respiration is comprised between 5% and 30% of their GPP	50% GPP < R <30% GPP	Vezina and Platt, 1988
	ph3	Upper and lower	ph3 respiration is comprised between 5% and 40% of their GPP	50% GPP < R < 40% GPP	Vezina and Platt, 1988
	bac	Lower	Bacteria respiration is at least 20% of their total uptake of doc	20% U_DOC_ < R	Vezina and Savenkoff, 1999
	hnf, mic mes	Lower	Zooplankton respiration is at least 20% of their total ingestion and doesn't exceed their maximum specific respiration	20% Σ Ing < R	Vezina and Savenkoff, 1999
	Chytrids	Upper	Sporangia and zoospores respiration doesn't exceed 20% of their carbon input	R < 20% C input	This study
Doc production	ph1, ph2 ph3	Upper and lower	Phytoplankton doc exudation is comprised between 10% and 55% of the net primary production (NPP)	10% NPP < E < 55% NPP	Breed et al., 2004
	hnf, mic mes	Upper and lower	Zooplankton exudation of doc is at least 10% of their total ingestion and doesn't exceed their respiration	10% Σ Ing < E < R	Vezina and Pace, 1994
Growth efficiency	hnf, mic mes	Upper and lower	The growth efficiency is no more than 50% of the total ingestion (Ing) and is at least 25% of it	25% Σ Ing < Ing − (R + E + Det) < 50% Σ Ing	Vezina et al., 2000
	bac	Upper and lower	Growth efficiency of bacteria is comprised between 25 % and 50%	0.5Σ Ing < R < 0.75Σ Ing	Vezina and Pahlow, 2003
Assimilation efficiency	hnf, mic mes	Upper and lower	Assimilation efficiency of zooplanctonic compartments is comprised between 50 % and 90% of their ingestion	50% Σ Ing < Ing -Det < 90% Σ Ing	Vezina et al., 2000
Grazing of ph3 by mes		Upper and lower	ph3 grazing by mes is comprised between 3% and 7% of its net primary production	3% NPP-ph3< Ing _ph3-mes_ < 7% NPP-ph3	Quiblier-Loberas et al., 1996
Predation on mic by mes		Upper	80% of total ingestion of mesozooplankton	Ing _mic-mes_ < 0.8 Σ Ing _mes_	Vezina et al., 2000
Preferential ingestion of mes	bac	Upper and lower	Bacteria consumption by mes is comprised between 10 and 15% of mes total ingestion	10%Σ Ing mes < Ing bac-mes < 15% Σ Ing mes	This study
	ph2	Upper and lower	ph2 grazing by mes is comprised between 10 and 15% of mes total ingestion	10%Σ Ing mes < Ing ph2-mes < 15% Σ Ing mes	
	hnf zsp	Upper and lower	The sum of hnf and zsp consumption by mes is comprised between 15 and 25% of mes total ingestion	15% Σ Ing mes< Ing hnf+zsp-mes < 25% Σ Ing mes	
	mic	Upper and lower	Predation of mes on mic is comprised between 40 and 60% of mes total ingestion	40%Σ Ing mes < Ing mic-mes < 60% Σ Ing mes	
Preferential ingestion of mic	bac ph1	Upper and lower	The sum of bac and ph1 consumption by mic is comprised between 10 and 15% of mic total ingestion	10%Σ Ing mic < Ing bac+ph1-mic < 15% Σ Ing mic	This study
	ph2	Upper and lower	ph2 grazing by mic is comprised between 20 and 30% of mic total ingestion	20%Σ Ing mic < Ing ph2-mic < 30% Σ Ing mic	
	hnf zsp	Upper and lower	The sum of hnf and zsp consumption by mic is comprised between 40 and 60% of mic total ingestion	40%Σ Ing mic < Ing hnf + zsp-mic < 60% Σ Ing mic	
Preferential ingestion of hnf	bac	Lower	bac consumption by hnf is at least 60% of hnf total ingestion	60% Σ Ing hnf < Ing bac-hnf	This study modified from Bettarel et al., 2003
	ph1	Lower	ph1 consumption by hnf is at least 20% of hnf total ingestion	20% Σ Ing hnf < Ing ph1-hnf	
Detritus production	hnf	Upper	hnf contribution to det carbon input doesn't exceed 20% of its total ingestion	hnf-det < 20% Σ Ing _hnf_	Carrias et al., 1998
	mes	Upper	mes contribution to det carbon input doesn't exceed 20% of its total ingestion	mes-det < 20% Σ Ing_mes_	
	bac	Upper and lower	Between 1.2% et 5.6% of bacterial production (BP) will contribute to the det carbon input (Attached bacteria)	1.2% BP < Bac -Det < 5.6% BP	Lemarchand et al., 2006
	ph3	Upper and lower	Microphytoplankton det production is comprised between 16% and 95% of total det production	16% Σ Det < ph3-det < 95% Σ Det	Arnous et al., 2010
	Chytrids	Upper	Det production by sporangia exceed 5% of its carbon input	Det _spg_ < 5% GPP3-spg	Niquil et al., 2011
Detritus consumption by mes		Upper	Mes consumption of detrital is no more than 40% of detritus production	Ing _det-mes_ < 40% Σ Det	This study
Detritus dissolution		Upper	The upper bound of det dissolution is 10% of net particular production	10% NPP < det-doc	Pace et al., 1984
Zoospores ingestion		Lower	Zoospora ingestion by mic is at least twice its ingestion by mes	Ing _zsp - mic_ > 2 Ing _zsp - mes_	This study
Carbon transfer from microphytoplankton to host-attached sporangia		Lower	The lower bound of carbon transfered to sporangia after infections of ph3 cells is 8% of net particular production	gpp-ph3 TO spg > 8% NPP-ph3	This study modified from Kagami et al., 2006
Carbon transfert from sporangia to zoospores		Lower	The lower bound of carbon transfered from sporangia to zoospores is at least the carbon biomass of zoospores compartment	spg TO zsp > Biom zsp	This study
Sinking/loss	ph3	Lower	ph3 sinking is at least 28% of total carbon sinking	ph3-los > 28% Σ los	Kagami et al., 2002
			Sedimentation of ph3 exceed 0.2 mgC m^−2^ d^−1^	ph3-los > 0.2	Carrias et al., 1998
	mes	Upper and lower	Sedimentation of mes range between 45% and 65% of total sedimentation	45% Σ loss < mes-loss < 65% Σ loss	Vezina et al., 2000

#### Solutions

The last step of the inverse analysis was the calculation of flows. A range of possible values was given by the method of the Monte Carlo Markov Chain joined to the mirror technique (Van Den Meersche et al., 2009). A jump value of 10 mgC m^−2^d^−1^ and 100,000 iterations were used to cover all possible solutions. More details on this method are available in Van Den Meersche et al. (2009) and Niquil et al. (2012).

### Ecological network analysis

The resulting flows issued from inverse analysis, together with estimated biomasses, were used for calculating Ecological Network Analysis indices in order to describe the emergent properties of the ecosystem.

*Total system throughput* (TST) represents a measure of the total system activity and is the sum of all the flows through all compartments (Kay et al., 1989).

*Average path length* (APL) is the average number of compartments crossed by a unit of carbon from its entry to the system to its leaving. It represents a measure of the system retention (Kay et al., 1989).

*System ascendency* (A) is a measure of the system size and organization. It is the product of the TST and the average mutual information (AMI: degree of specialization of flows in the network) (Ulanowicz, 1986). This value is more informative about the organization of the system when it is expressed in relation to development capacity and considered as its maximum value (A/DC). It defines the ecosystem degree of development. High Relative ascendency indicates more specialized and less redundant pathways. The structure of energy flows can be related to the concept of structural asymmetry and ecosystem stability (Rooney et al., 2006), because the pattern of asymmetric channel flow enhances the equilibrium stability of an ecosystem. The difference between the development capacity and the ascendency is called redundancy (R), which is a quantification of the multiplicity of parallel flows. The relative redundancy (R/DC, %) is a measure of the ecosystem degree of information loss due to parallel pathways. The system would be redundant when the ascendency is low.

*Development capacity* (DC) is calculated as the product of TST and the upper limit of AMI, corresponding to the maximum potential ascendancy and to a food web with maximum specialization. The development capacity is the sum of ascendency, redundancy and information loss related to external exchanges.

In this study, as suggested by Ulanowicz (1986), growth and development were characterized by indices calculated over only internal exchanges. We consider the internal capacity of ecosystem development (DCi), i.e., the sum of internal ascendency (Ai) and internal redundancy (Ri). The internal relative ascendency Ai/DCi could point to a strong dependency of an ecosystem on external inputs (Baird and Heymans, 1996) in case it decreases in relation to the A/DC ratio. However, as pointed out by Baird et al. (1991), Ai/DCi ratio could be an aspect of a highly organized ecosystem. The internal relative redundancy (Ri/DCi) is considered as a measure of ecosystem stability by many authors (Rutledge et al., 1976; Baird et al., 1998, 2004).

*Finn Cycling Index* (FCI) is the ratio of carbon flowing in loops (the carbon comes back to the compartment it left) to the sum of all carbon flows. I.e., it is the fraction of all flows involved in recycling (Finn, 1976) and can also be considered a measure of the retentiveness of a system.

*Connectance* measures the trophic connections within a food web. The overall connectance includes the effects of all transfers (exogenous and endogenous exchanges). The intercompartmental connectance characterizes only the endogenous exchanges. The food-web connectance pertains only to transfers among the living compartments (Ulanowicz, 2003).

*Comprehensive Cycling Index* (CCI) was proposed as a new index that gives the real importance of cycling after added corrections to the FCI. This new index considers four types of pathways that energy and matter can follow to join one compartment to another (Allesina and Ulanowicz, 2004).

*Trophic analysis* maps the complex network of trophic transfers as a linear food chain (called Lindeman spine, Ulanowicz and Kemp, 1979) based on the trophic concept of Lindeman (Lindeman, 1942). The Lindeman spine allows calculation of the trophic efficiency for each level, also called transfer efficiency (Ulanowicz and Wulff, 1991). The global trophic efficiency (Geff) is computed as the logarithmic mean of all the trophic level efficiencies. Two more indices were derived from the Lindeman spine: the grazing chain efficiency and the percentage of detritivory. The mean values of the 100,000 set of flows resulting from the LIM-MCMC analysis were used to build the Lindeman spine using the WAND software.

*Detritivory/Herbivory* ratio (D/H) is the sum of consumption of non-living material (detritus or DOC) divided by the sum of flows of consumption of autotrophic organisms. Parasitism was included in herbivory flow.

For these calculations, we used an algorithm written for Matlab^©^ by Carole Lebreton and Markus Schartau (pers. comm.) to calculate the first set of indices: TST, APL, A, DC, R, Ri and connectance. 100,000 iterations of these ecological indices were computed allowing statistical analysis of the difference between those calculated for Lake Pavin and Lake Aydat. Recycling, connectance and trophic efficiencies indices were calculated using the ecological network analysis package WAND^©^ by Allesina and Bondavalli (2004) available at http://www.dsa.unipr.it/netanalysis/?Software. Only one value index was obtained for these indices, based on the average flow value for each flow of the food-web, preventing us from statistically testing the difference between the two blooms.

### Data analysis

The statistical comparison between the two situations described, concerning the set of indices calculated under Matlab (TST, APL, A, DC, R, Ri and connectance) was tested with two tailed Student (t) tests using XLSTAT^©^.

## Results

### Flow analysis

**Figure 1:**
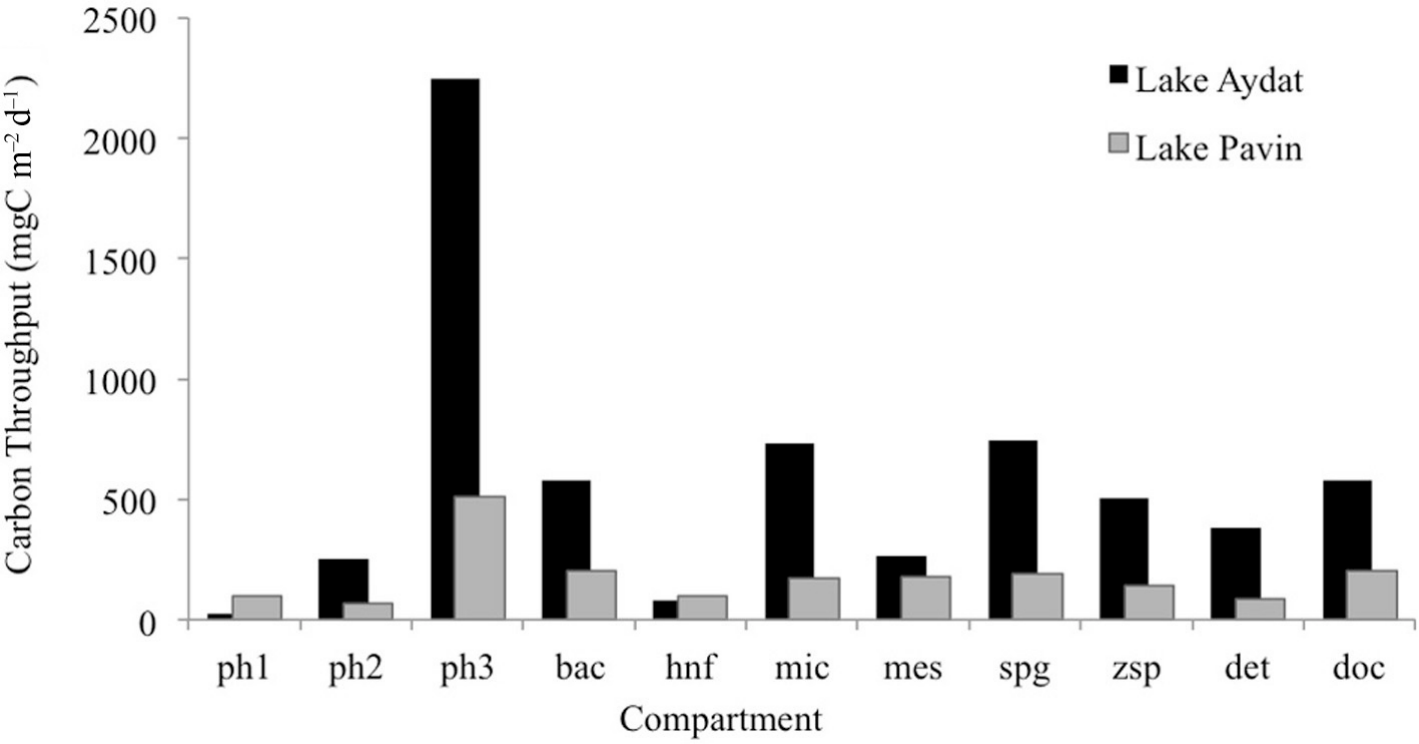
**Throughput of each compartment for both models (mgC m^−2^ d^−1^): Pavin Spring bloom model (white chart) and Aydat Autumn bloom model (black chart)**. Bac, bacteria; Ph1, picophytoplankton; Ph2, nanophytoplankton; Ph3, microphytoplankton; Hnf, heterotrophic nanoflagellate; Mic, microzooplankton; Mes, mesozooplankton; Spg, sporangia; Zsp, zoospores; Det, detritus; Doc, dissolved organic carbon.

The values for overall flows for both models are given in Table 3. Carbon input into the lakes was only due to primary production. No allochthonous detritus input was considered due to the small catchment area (50 ha) of Lake Pavin and the low rainfall during the study period in Lake Aydat. Due to blooms of large algae in Lake Pavin (*Synedra* sp., *Melosira* sp., *Cyclotella* sp., and *Asterionella* sp.) and filamentous cyanobacteria in Lake Aydat (*Anabaena* sp. and *Oscillatoria* sp.) the major contribution to the gross primary production was provided by microphytoplankton (75% and 89% in Lake Pavin and Aydat, respectively). Calculated carbon throughput (T) of microphytoplankton was higher in Lake Aydat (2243 mgCm^−2^d^−1^). Consequently, all throughputs (and primarily those directly affected by primary production) were more important in Lake Aydat (Figure 1). According to the trophic status of the lakes, the contribution of picophytoplankton production to total gross primary production was higher in the oligo-mesotrophic Lake Pavin (14.7%) compared to the eutrophic Lake Aydat (1%).

**Table 3:** **Flow description, name and corresponding value (mg C m^−2^ d^−1^) of steady state models of the pelagic food web of Lake Pavin and Lake Aydat during each lake bloom period**.

**Flow description**	**Flow name**	**Pavin spring bloom**	**Aydat Autumn bloom**
		**Inferred value (mg C m^−2^ d^−1^)**	
		**Model I**	**Model II**
Microphytoplankton gross primary production	gpp-ph3	*509.35*	*2243.38*
Nanophytoplankton gross primary production	gpp-ph2	*66.62*	*253.91*
Picophytoplankton gross primary production	gpp-ph1	*100.28*	*22.70*
Microphytoplankton respiration	ph3-res	192.25	112.62
Microphytoplankton doc excretion	ph3-doc	39.10	193.94
Microphytoplankton grazing by mic	ph3-mic	13.25	0.26
Microphytoplankton grazing by mes	ph3-mes	11.89	64.05
Parasitism of ph3 by sporangia	ph3-spg	*187.44*	*746.00*
Microphytoplankton det production	ph3-det	15.50	228.93
Microphytoplankton sinking	ph3-los	*49.92*	897.58
Nanophytoplankton respiration	ph2-res	4.29	15.42
Nanophytoplankton doc excretion	ph2-doc	6.63	24.20
Nanophytoplankton grazing by mic	ph2-mic	35.74	184.98
Nanophytoplankton grazing by mes	ph2-mes	18.53	26.69
Nanophytoplankton sinking	ph2-los	*0.74*	2.48
Nanophytoplankton det production	ph2-det	0.70	0.14
Picophytoplankton respiration	ph1-res	19.86	2.58
Picophytoplankton doc excretion	ph1-doc	23.92	3.16
Picophytoplankton grazing by hnf	ph1-hnf	34.57	16.53
Picophytoplankton grazing by mic	ph1-mic	16.60	0.18
Picophytoplankton grazing by mes	ph1-mes	5.34	0.26
Bacteria respiration	bac-res	98.85	327.02
Bacterivory by hnf	bac-hnf	66.39	*63.02*
Bacteria uptake by mes	bac-mes	20.74	26.63
Bacteria uptake by mic	bac-mic	6.47	*109.40*
Bacterial doc release due to viruses lysis	bac-doc	9.90	0.71
Attached bacteria to det	bac-det	2.09	44.00
Heterotrophic nanoplankton respiration	hnf-res	38.12	27.37
Heterotrophic nanoplankton doc excretion	hnf-doc	21.56	14.55
Heterotrophic nanoplankton uptake by mic	hnf-mic	13.97	14.74
Heterotrophic nanoplankton uptake by mes	hnf-mes	14.65	14.78
Heterotrophic nanoplankton det production	hnf-det	12.66	8.11
Microzooplankton respiration	mic-res	47.82	255.55
Microzooplankton doc excretion	mic-doc	29.85	145.81
Microzooplankton uptake by mes	mic-mes	73.64	105.98
Microzooplankton egestion	mic-det	19.85	73.33
Microzooplankton sinking	mic-los	2.54	151.10
Mesozooplankton respiration	mes-res	48.57	93.11
Mesozooplankton doc excretion	mes-doc	30.49	50.08
Mesozooplankton egestion	mes-det	20.29	26.57
Mesozooplankton grazing by larger organisms	mes-los	78.64	94.42
Sporangia respiration	spg-res	32.93	74.86
Sporangia emission of zoospores	spg-zsp	*140.45*	*504.12*
Sporangia detrital production	spg-det	3.75	0.15
Sporangia sinking	spg-los	10.31	166.86
Zoospores respiration	zsp-res	24.46	56.94
Zoospores ingestion by mic	zsp-mic	87.68	421.87
Zoospores ingestion by mes	zsp-mes	19.92	25.15
Zoospores detrital production	zsp-det	8.40	0.15
Dissolved organic carbon uptake by bacteria	doc-bac	204.45	570.78
Detritus dissolution	det-doc	43.00	138.34
Detritus consumption by mes	det-mes	13.29	0.65
Detritus consumption by mic	det-mic	-	0.35
Detritus sinking	det-los	26.95	242.06

Italic values indicate flows that were estimated or derived from processes determined in situ. The rest are values constrained by one or two inequations and estimated by the LIM-MCMC method.

**Figure 2:**
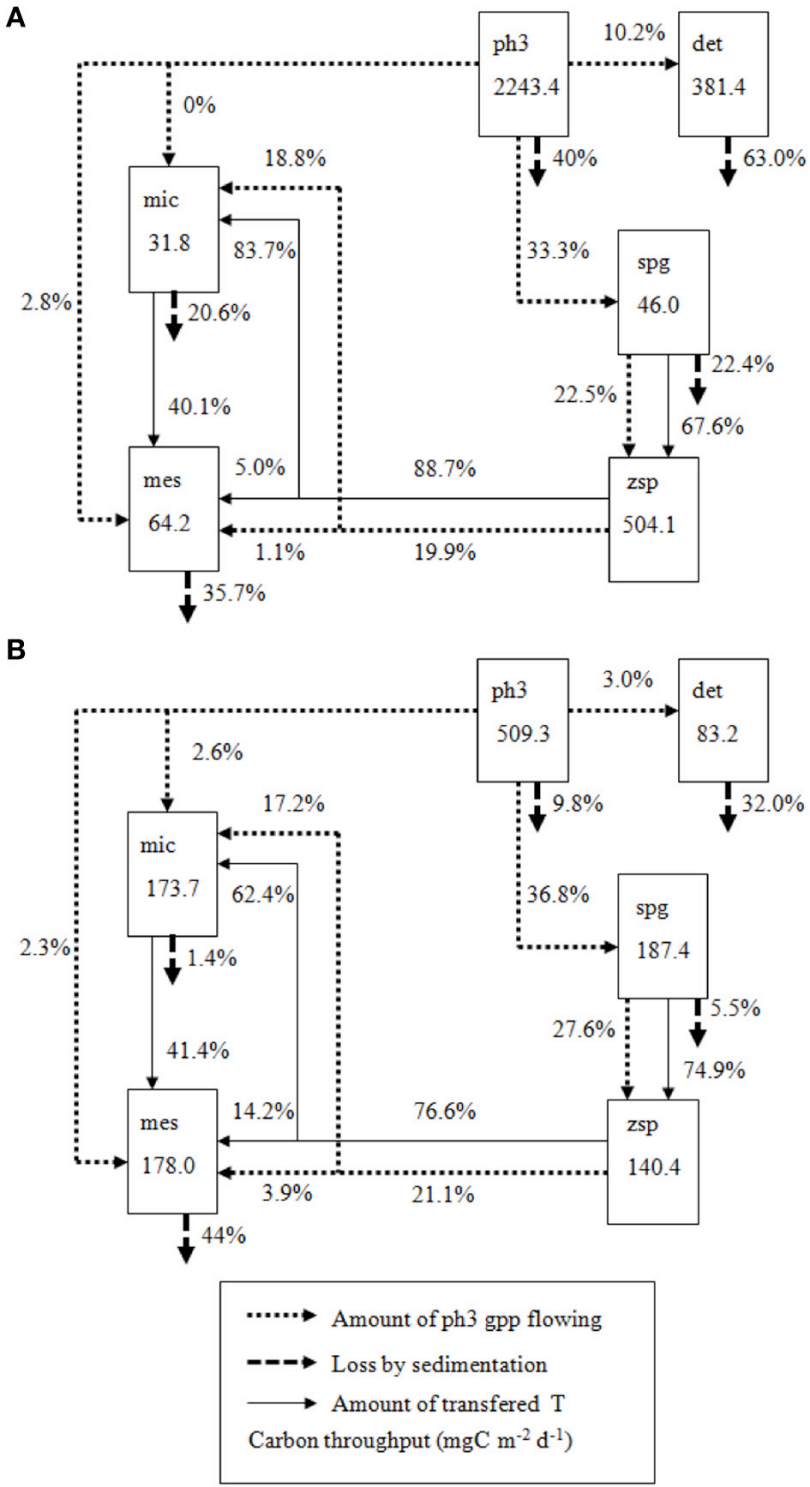
**Carbon sinking and flowing from ph3 compartment to the other compartments with highlights on carbon involved in chytrids for (A) Pavin bloom model and (B) Aydat bloom model**. Bac, bacteria; Ph1, picophytoplankton; Ph2, nanophytoplankton; Ph3, microphytoplankton; Hnf, heterotrophic nanoflagellate; Mic, microzooplankton; Mes, mesozooplankton; Spg, sporangia; Zsp, zoospores; Det, detritus; Doc, dissolved organic carbon.

The flows calculated in mgC m^−2^d^−1^ by the LIM—MCMC method directly involved in fungal compartments are detailed in Figure 2. In Lake Pavin, 9.6% of total system throughput was channeled to the sporangia parasitic compartment, which corresponded to 36.8% of microphytoplankton production. The sporangia compartment channeled 75% of its carbon input to the zoospore compartment. In Lake Aydat, the calculated percentage of the total system throughput values channeled to the sporangia compartment was slightly higher than in Lake Pavin and represented 11.7% (33% of *ph3* gross primary production). However, the sporangia compartment channeled less carbon to the zoospores compartment (67.6%). This was linked to a major sporangia loss flow in Lake Aydat (22.4% of total sporangia throughput) compared to Lake Pavin (only 5.5%).

**Figure 3:**
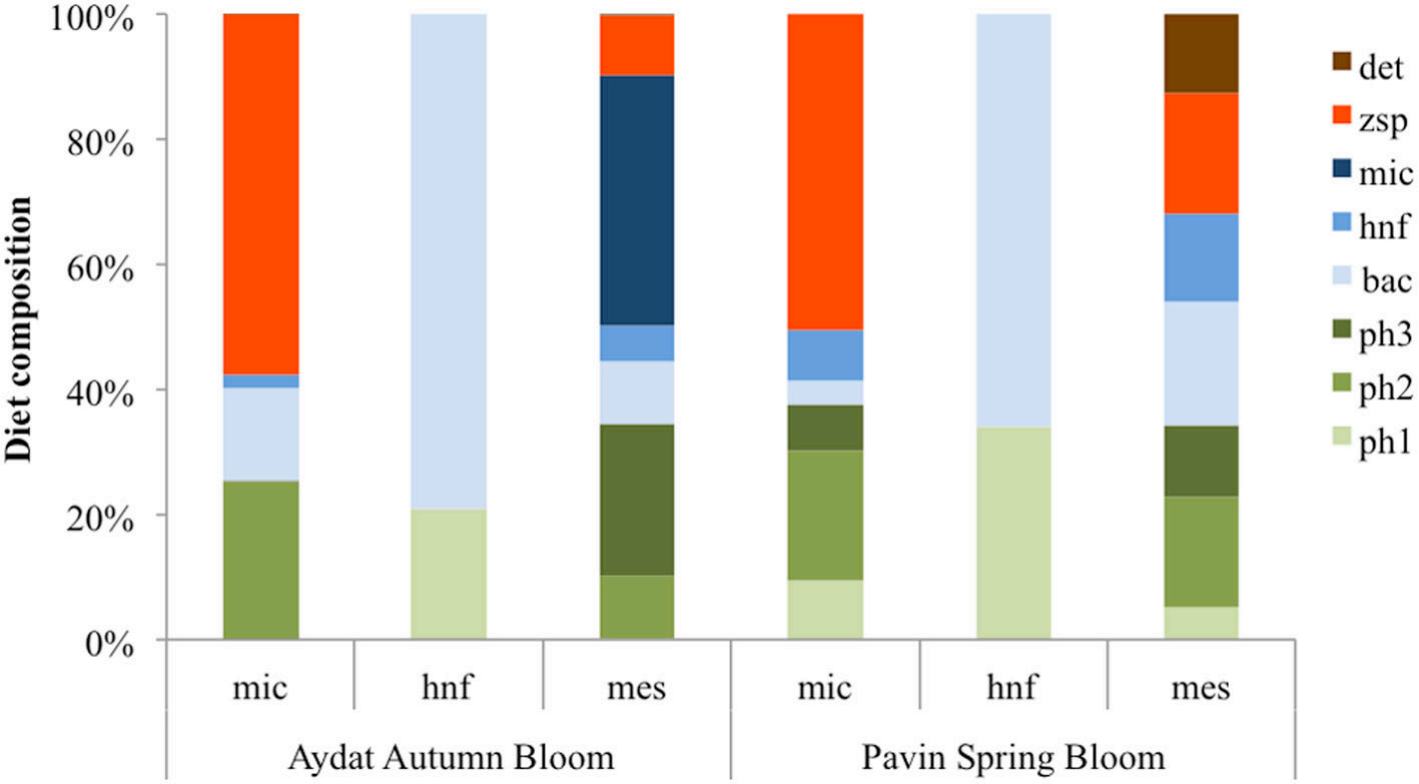
**Diet composition of each grazer for each Lake**. Bac, bacteria; Ph1, picophytoplankton; Ph2, nanophytoplankton; Ph3, microphytoplankton; Hnf, heterotrophic nanoflagellate; Mic, microzooplankton; Mes, mesozooplankton; Spg, sporangia; Zsp, zoospores; Det, detritus; Doc, dissolved organic carbon.

Carbon flows involved in parasitism were of major importance for grazer compartments (i.e., micro- and mesozooplankton). Major zoospore throughput was channeled to the microzooplankton compartment (62.4% and 83.7%, respectively for Pavin and Aydat) and only 14.2% and 5% of zoospore throughput was ingested by the mesozooplankton compartment (Figure 2). Zoospores constituted the major nutritional resource for microzooplankton in Lake Pavin (50.5%) and in Lake Aydat (57.7%) (Figure 3). However, since microzooplankton represented the main ingested prey by mesozooplankton in both lakes (about 40–41%), carbon that originated from microphytoplankton parasitism was indirectly channeled to mesozooplankton through its ingestion of major microzooplankton throughput (Figure 2).

Moreover, carbon indirectly channeled from microphytoplankton to grazers through ingestion of zoospores produced by parasites' sporangia, represented 19–21% of microphytoplankton gross primary production in both lakes (Figure 2). This corresponded to 57–60% of sporangia throughput transferred to grazer compartments.

Sedimentation flows were lower in Lake Pavin compared to Lake Aydat, and represented 25% and 61.7% of the total system throughput, respectively. The largest contribution to this loss flow in Lake Aydat originated from microphytoplankton (57.7%, Table 4) while it originated from mesozooplankton (46.5% of total system loss, Table 4) in Lake Pavin. Detritus production originated mainly from microphytoplankton in the Aydat ecosystem (60% of total detritus throughput, Table 4) while in Lake Pavin zooplankton was the main contributor (48.2%, Table 4).

**Table 4:** **Percentage of total carbon throughput loosed by sedimentation and contribution to detritus throughput of each compartment and for both Lakes**.

	**Pavin spring bloom**		**Aydat Autumn bloom**	
	**Loss**	**det**	**Loss**	**det**
ph1	0.00	0.00	0.00	0.00
ph2	0.44	0.84	0.16	0.04
ph3	29.52	18.62	**57.74**	**60.02**
bac	0.00	2.52	0.00	11.54
hnf	0.00	15.21	0.00	2.13
mic	1.50	**23.85**	9.72	19.23
mes	**46.50**	**24.38**	6.07	6.97
zsp	0.00	10.09	0.00	0.04
spg	6.10	4.50	10.73	0.04
det	15.94	0.00	15.57	0.00

Bold indicates higher values.

### Ecological network analysis

The LIM-MCMC derived flow results of each model were used to calculate the ecological network indices. Table 5 provides comparisons of the most relevant ecological network analysis indices for both lakes. Significant differences (*P* < 0.05) in network properties of the two considered webs were observed for TST, DC, A, A/DC, DCi, Ai and Ri. No relevant significant difference was detected between both lakes for APL, AMI, R/DC, Ai/DCi and Ri/DCi.

**Table 5:** **Topological indices of pelagic food webs of Lake Pavin Spring bloom and Lake Aydat Autumn bloom; and *t* test results**.

	**Abv**	**Lake pavin**	**Lake aydat**	**Test *t p* value**	**Alpha 0.05**
Total system throughput	TST	2625	**8889**	<0.0001	^**^
Average path length	APL	**2.9**	2.5	0.056	ns
Development capacity	DC	8420	**26,054**	<0.0001	^**^
Ascendency	A	5134	**17,287**	<0.0001	^**^
Relative ascendency	A/DC	61%	**66%**	0.039	^*^
Average mutual information	AMI	**1.96**	1.94	0.82	ns
Relative redundancy	R/DC	**47%**	45%	0.125	ns
Internal capacity	DCi	7097	**21,472**	<0.0001	^**^
Internal ascendency	Ai	3109	**9650**	0	^**^
Internal redundancy	Ri	3988	**11,821**	<0.0001	^**^
Internal relative ascendency	Ai/DCi	44%	**45%**	0.542	ns
Internal relative redundancy	Ri/DCi	**56%**	55%	0.5	ns

Bold indicates higher values.

* Alpha < 0.05;

** Alpha < 0.01.

The total system throughput (TST, Table 5) was much lower in Lake Pavin compared to Lake Aydat due to higher primary production and carbon input during the cyanobacteria bloom. The calculated averages were 2625 and 8889 mgCm^−2^d^−1^, respectively, for Pavin and Aydat.

The average mutual information (AMI) values gives information about flow specialization. Values of AMI were almost the same for Aydat and Pavin (1.94 for Aydat and 1.96 for Pavin).

The average pathway length (APL), a measure of the system retention capacity, was not significantly higher in Lake Pavin (Table 5). The calculated mean number of compartments through which each inflow passes was 2.9 in Lake Pavin and 2.5 in Lake Aydat.

The topological indices, which characterize the ecosystem structure, were affected by the different carbon input in different trophic situations of the two lakes and were lower in Lake Pavin compared to Lake Aydat except for redundancy. The development capacity was higher for Lake Aydat (Table 5) following the pattern of higher TST due to cyanobacteria bloom. Relative ascendency (A/DC%) also was affected by higher TST in the eutrophic lake, being around 66% in Aydat and 61% in Pavin. System inefficiency due to internal parallel pathways, termed relative redundancy (R/DC), was higher, but not significantly, in Lake Pavin during the spring bloom (Table 5). Internal indices calculated over only internal exchanges showed higher internal ascendency (Ai) and internal redundancy (Ri) for the Lake Aydat, due to the presence of populations more adapted to rapid changes and the effect of parasites helping to restablish broken pathways more easily. But, relative internal or functional redundancy (Ri/DCi) was slightly more important for Lake Pavin (56%) due to the similar effect of parasites over the internal exchanges.

The overall connectance, number of trophic connections, within each food web including exogenous transfers, was higher for Lake Pavin (2.2 vs. 1.9) due to the effect of parasites in reducing the losses of organic matter and enhancing the transfers through the mycoloop. Even when only based on endogenous exchanges, the amount of trophic connections given by the inter-compartmental connectance index was also higher in the Lake Pavin ecosystem (2.3 vs. 1.9). The same was true for food web connectance, which pertains only to transfers among living compartments (1.8 vs. 1.5).

The Finn Cycling Index (FCI), showed higher recycling activity through the Lake Pavin (6%) than the Lake Aydat (3%) food web due to the higher exploitation of zoospores energetic by higher trophic levels and stronger loops. CCI, an update of the FCI index, also showed a higher recycling for Lake Pavin compared to Lake Aydat (7% vs. 4%).

All trophic level efficiencies derived from the Lindeman spine analysis were higher in Lake Pavin with the exception of trophic level III, which was more efficient in carbon transfer in Lake Aydat (66.10%) than Lake Pavin (48.33%) (Table 6). Trophic Level III was represented by zoospores, micro- and mesozooplankton. The global trophic efficiency (Geff) index confirmed that carbon was better transferred from the lowest to the highest trophic levels in Lake Pavin (47.09%) (Table 6). The Geff index is linked to grazing chain efficiency.

**Table 6:** **Indices derived from the Lindeman spine**.

	**Pavin spring bloom**	**Aydat autumn bloom**
**TROPHIC LEVEL (TL) EFFICIENCY (%)**		
1st TL	**64.82**	56.03
2nd TL	**50.18**	45.60
3rd TL	48.33	**66.10**
4th TL	**31.29**	12.90
Global trophic efficiency	**47.09**	38.42
**GRAZING CHAIN EFFICIENCY (%)**		
1st TL	**80.04**	64.08
2nd TL	**40.17**	29.22
3rd TL	**19.41**	19.31
4th TL	**6.07**	2.49
**DETRITIVORY/HERBIVORY**		
D/H	**67.44**	55.44

Bold indicates higher values.

The Lake Pavin network exhibited a lower rate of detritivory (219 vs. 576 mgC m^−2^d^−1^) and circulation within the detrital pool (42.98 vs. 144.68 mgC m^−2^d^−1^) compared to Lake Aydat. However, the D/H ratio was higher in Lake Pavin than in Lake Aydat (Table 6).

## Discussion

### Parasites and algal blooms

The planktonic food webs modeled in our study are considered to be representative of intensive algal proliferation (bloom) situations in two contrasting freshwater ecosystems. Lake Pavin is characterized by the dominance of diatoms during spring blooms while Lake Aydat shows a dominance of cyanobacteria during autumn blooms. In the classical food web version with no parasites, phytoplankton biomass produced during such proliferation events were considered to be lost by sinking (Malone, 1980; Legendre and Le Fevre, 1991; Kiorboe, 1993), and thus not able to sustain higher trophic levels. Some blooming species are even considered to be toxic, leading to reproductive failure in marine and freshwater invertebrates, interfering with zooplankton feeding (Rohrlack et al., 1999) and limiting their distribution (Ianora and Miralto, 2010). A study on the feeding behavior of copepods during phytoplankton spring blooms showed that non-phytoplankton prey supported 40–71% of the copepod carbon requirement (Kobari et al., 2010).

However, it has been shown that some mesozooplankton (i.e., Cladocera) coexist with blooming filamentous cyanobacteria (Epp, 1996), and phytoplankton parasites can constitute an important food source during such situations. Indeed, fungal life stages could represent key intermediates in the pelagic food chain (Gleason et al., 2009). Chytrid zoospores were found to be efficiently grazed by mesozooplankton (Kagami et al., 2004) and could even sustain *Daphnia* growth cultured *in vitro* (Kagami et al., 2007). There has been evidence for higher instances of parasitism leading to less ecosystem reliance on detritus consumption and its related recycling (Niquil et al., 2011).

Our study focused on the algal bloom peak episodes in Lakes Pavin (from 4 to 18 April 2007) and Aydat (from September 24 to October 10 2007) and complements previous work conducted during the spring epidemic of chytrid parasites (whole spring diatom growth, from March to June 2007) in Lake Pavin (Grami et al., 2011), which found that parasitism indirectly channels primary produced carbon to grazers and that this process is an important part of the food web functioning. We were able to show the importance of parasites as trophic links, the importance of parasitic zoospores as nutritional sources able to sustain zooplankton diet, and their role in supporting upper trophic levels and recycling algal biomass.

### Carbon flows involved in parasitism

Parasitism affected the flow of carbon during the diatom peak bloom in Lake Pavin (676.25 mgC m^−2^ d^−1^) and the cyanobacteria peak bloom in Lake Aydat (2520 mgC m^−2^ d^−1^). Here, total gross primary production in both lakes was higher than reported in the previous model constructed for Lake Pavin during a longer period (Grami et al., 2011; 360.54 mgC m^−2^ d^−1^, March to June 2007). These values corroborate previous findings on the trophic status of both an oligo-mesotrophic lake (Amblard et al., 1992) and a eutrophic lake (Aleya et al., 1988). These values are consistent with gross primary production measured during a study conducted during spring phytoplankton proliferation in the north basin of the mesotrophic Japanese Lake Biwa (1639 mgC m^−2^ d^−1^; Yoshimizu et al., 2001).

A large amount of the dominant primary producer's carbon (microphytoplankton) was involved in sporangia development (37% and 33%, respectively, in Lake Pavin and Lake Aydat); representing 28% and 29% of their total primary production. During both blooms the planktonic community was characterized by large inedible algae, high biomass and high gross primary production (*ph3* GPP). Under these circumstances, the channeling of primary production through the food web should be highly impacted by the presence of chytrids. Values of the highest possible impact of chytrids given by model simulations of different rates of parasitism on microphytoplankton tested in the Lake Biwa by Niquil et al. (2011), were around 35% of microphytoplankton GPP.

Grazers were found to be indirectly sustained by primary production through the consumption of parasitic zoospores. Indeed, a large percentage of the throughput originating mainly from sporangia was channeled to microzooplankton (76% and 83% for Lake Pavin and Lake Aydat, respectively). Values based on the mean spring model of Lake Pavin (Grami et al., 2011), were lower: only 21% of the microphytoplankton production was channeled through chytrids sustaining 38% of microzooplankton diet. Considering the higher primary production input during the bloom period, we compared our results with those obtained from a modeling study of Lake Biwa (Niquil et al., 2011). The primary production rate was around 1580 mgC m^−2^ d^−1^ and the estimated chytrid contribution to the diets of microzooplankton was only 10%; bacterial prey were the main carbon source (70% of their diet, Niquil et al., 2011). In a previous study, Nakano et al. (1998) reported a low ingestion rate of HNF during summertime. This confirms our hypothesis that chytrid spores could have been previously misidentified in the flagellate community and therefore have a high potential as a trophic link. During the bloom they are able to replace bacterivory and lead a higher recycling of large phytoplankton biomass.

Estimation of microbial food web efficiency (microzooplankton efficiency; 36.7% vs. 14.4%) and the microbial link (percentage of mesozooplankton demand; 53% vs. 50%), showed higher values for Lake Pavin compared to Lake Aydat. Microzooplankton efficiency, which was significantly higher for Lake Pavin, represents carbon transfer efficiency from micro- to mesozooplankton (Gaedke and Straile, 1994) calculated as microzooplankton ingestion by mesozooplankton divided by microzooplankton throughput. Based on this percentage, we could conclude that Lake Pavin, due to its higher primary production, transferred to sporangia *via* parasitism (36.8% vs. 33.25%) then to zoospores (74.86% vs. 67.6%) possessed a higher microbial food web efficiency than Lake Aydat. Comparing our models with models from the literature reveals that Lake Pavin, which is characterized by large inedible algae with high parasitism of the dominant species, has similar values to environments characterized by primary production, mainly from edible picophytoplankton (as estimated by Niquil et al., 2001 in the Takapoto atoll). Therefore, based on microzooplankton efficiency and microbial link values (Table 7), parasites are able to replace inedible phytoplankton, thus providing edible resources that appear to be sufficient to meet zooplankton energy requirements and sustain the carbon demand of higher trophic levels.

**Table 7:** **Comparison of some food web indicators (main contributors to PP, Microbial food web efficiency, Microbial Link and trophic efficiency at Level II) calculated for Lake Pavin and Lake Aydat and other ecosystems**.

	**Lake Biwa**	**Lake Kinneret**	**Takapoto atoll**	**English channel**	**Celtic sea**	**Aydat lake**	**Pavin lake**
References	Niquil et al., 2011	Stone et al., 1993	Niquil et al., 2001	Vezina and Platt, 1988	Vezina and Platt, 1988	This study	This study
Main contributor to primary production (% of GPP)	phyto. > 20 μm (70%)	Non-pyrrophytes (90%)	phyto. < 3 μm (74%)	ND	ND	phyto. > 20 μm (89%)	phyto. > 20 μm (75%)
Microbial food web efficiency or microzooplankton efficiency (Mic-mes/mic throughput) (%)	10	26	38	37	46	14.40	36.70
Microbial Link as % of mes demand (mic-mes+ bac-mes/mes throughput) (%)	5.50	16	29	23	20	50	53
Trophic efficiency at Level II (%)	16	38	27	41	32	45.60	50.18

Parasitism could allow a better carbon transfer from primary producers to zooplankton. This pathway (producers > parasites > grazers), called the “Mycoloop” by Kagami et al. (2007), was quantified in this study during the particularly large algae proliferation. The presence of parasites allowed not only a recycling of the high input of phytoplankton biomass through an increase of energy transfer from primary producers to consumers, but also a consequent overcoming of the trophic bottleneck created by the large amount of inedible biomass in the lake.

The mycoloop may also have an impact on sedimentation rates, which are usually very important during an inedible algal bloom. When considered for the first time in a pelagic food web model, parasitism induced less carbon loss from the pelagic zone by reducing direct sedimentation of large phytoplankton species (21–10% of *ph3* GPP) and their detritus production (11–3% of their *ph3* GPP) (Pavin spring model, Grami et al., 2011). During the two bloom situations, Lake Pavin had a lower percentage of carbon loss by sedimentation from the microphytoplankton compartment (9.8% vs. 40% of microphytoplankton GPP), lower detritus production by *ph3* (3% vs. 10.2% of microphytoplankton GPP) and better carbon transfer from microphytoplankton to sporangia (36.8% vs. 33.3%). Some studies observed that during a bloom 40–60% of the cells reaching the hypolimnion were still viable (Amblard and Bourdier, 1990). It would therefore appear that in the absence of parasites the majority of algal production is lost by sinking and is unavailable to support higher trophic levels. In the eutrophic Lake Aydat, the cyanobacteria production was so high that even parasitism could not help channel high rates of this carbon to higher trophic levels (i.e., zooplankton) and the carbon loss due to sedimentation of microphytoplankton still accounted for 57.7% of the total. In oligo-mesotrophic Lake Pavin, parasitism helped to reduce carbon sedimentation loss to only 9.8%, during the peak of spring diatoms, 20% of algal production was channeled to higher trophic levels and lost afterwards by sinking of the large grazers.

### Ecosystem emergent properties

This paper sets out, for the first time, a model that explicitly investigates the influence of parasitism on pelagic food web properties under algal bloom situations in two different lake ecosystems. Indeed, using the LIM-MCMC method, we quantified and compared the amount of carbon that reached high trophic levels *via* the mycoloop.

Past studies have investigated the impact of parasites on ecosystem properties (Huxham et al., 1995; Thompson et al., 2005; Lafferty et al., 2008; Sato et al., 2011). If high relative ascendency (A/DC) ratios reflect high degrees of organization (Ulanowicz, 1986), then, in the presence of parasites, Lake Aydat is slightly more organized than Lake Pavin (66% vs. 61%, significant difference at *P* < 0.05). However, due to redundancy, the ecosystem structure can evolve to counter the effects of external perturbations (Ulanowicz, 2003). In this case, Lake Pavin should be only slightly more resistant to stress than Lake Aydat. Ulanowicz et al. (2009) stated that the balance between the organized and non-organized parts of ecosystems increases resistance and stability; Heymans et al. (2002) suggested that redundancy should increase ecosystem resilience. If so, Lake Pavin and Lake Aydat should be equally stable and resilient.

Ascendency combines the total system activity (TST), with the organization by which the component processes are linked (AMI). It gauges how well the system is performing at processing the given medium. Chytrids were found to allow a higher fraction of the total system throughput to pass along specialized pathways (Grami et al., 2011). The degree of specialization measured by the AMI index (Ulanowicz, 1997) gave almost no difference between Lake Aydat and Lake Pavin during blooms, suggesting that both lakes have equally specialized pathways.

Baird and Ulanowicz (1993) considered the internal ascendency indices (Ai/DCi), calculated over only internal exchanges, as an aspect of a highly organized ecosystem. Given that, Lake Aydat and Lake Pavin, with almost equal Ai/DCi index, might have the same tendency to internalize their activity. Since a relevant decrease was observed for the Ai/DCi ratio in relation to the A/DC ratio for both lakes, it could point to a strong dependency of Lake Pavin and Lake Aydat on external inputs (Baird and Heymans, 1996). However, Rutledge et al. (1976) and Baird et al. (1998, 2004) considered the internal redundancy (Ri/DCi) as a measure of ecosystem stability. If so, with no significant difference between values of Ri/DCi given for Lake Pavin and Lake Aydat, it seems that broken pathways might be equally re-established for both, which would result in the same level of stability and resilience.

Parasites are known to add links to food webs (Lafferty et al., 2008), as observed in estuarine (Lafferty et al., 2005; Thompson et al., 2005; Kuris et al., 2008) and freshwater (Kudoh and Tokahashi, 1990; Sukhdeo, 2010; Amundsen et al., 2013) ecosystems. Even if this seems logical, as for the addition of any species to a food web, this is especially true for parasites that have a free-living stage that can interact with other compartments and that may be eaten (Gross et al., 2009), such as chytrids zoospores. Parasitism also enhances the number of flows (Grami et al., 2011) and the number of compartments through which each inflow passes. Lake Pavin, with a slightly higher average path length (APL) value than Aydat (Table 5), has a longer chain length. This important structural property defines the number of links in a food chain and the energy transfer through its components (Jordan et al., 2003). According to Neutel et al. (2002), parasites with their complex life cycles, have more carbon loops with low interactions, which can offset the destabilizing effect of higher connectance. Lafferty et al. (2008) found that parasitism enhances connectance two fold. Chytrids in the Lake Pavin increased slightly the number of links and enhanced especially the inter-compartmental connectance compared to Lake Aydat, which represents the robustness of interactions inside an ecosystem, a measure of its organization. Robustness, indeed, is considered as a parameter linked to the stability of an ecosystem.

Cycling helps ecosystems optimize exploitation of resources by allowing a better use of energy and matter introduced into the system. In an ecosystem with low cycling (calculated by FCI), loss of carbon is thought to be higher, such as in the case of Lake Aydat compared to Lake Pavin. When perturbations occur, cycling reduces impacts to the ecosystem by acting as a buffer against large changes and can increase the ability of the ecosystem to resist changes. Then, the higher FCI in Lake Pavin increases the residence time of carbon within the ecosystem, making it more resistant to change. Another simple and effective measure of the quantitative importance of cycling in ecosystems is the comprehensive cycling index (CCI), which is one of the principal ways that ecosystem complexity can enhance stability (Allesina and Ulanowicz, 2004). CCI was also higher in the Lake Pavin ecosystem. Odum (1969) had identified the amount of cycling as one of his 24 criteria for “mature” or developed ecosystems. Wulff and Ulanowicz (1989) suggested that the increased amount of cycling is a homeostatic response of the ecosystem to stress.

## Conclusions

To our knowledge, field data on pelagic ecosystem trophodynamics during bloom events with and without parasites do not yet exist. Most models use the basic tenet “zooplankton eat phytoplankton” and parasites have long been considered as playing an insignificant role in ecosystem steady-state functioning. Our models document the carbon transfer channeled by parasites from primary producers to consumers in two ecosystems dominated by inedible phytoplankton biomass and production. We provided quantitative estimates of the importance of parasitism as an indirect pathway channeling primary production contained in “inedible algae” and showed that this primary-produced carbon is efficiently conveyed to grazers and able to sustain zooplankton during inedible algal blooms, especially in the oligomesotrophic Lake Pavin. By considering parasites, our study indicates that the ecosystem becomes more efficient and specialized, and less reliant on detritivory, which is consistent with various suggestions about the influence of parasitism on ecosystem properties. In short, parasitism on phytoplankton stabilizes the system significantly during inedible algal blooms, especially in the case of oligo-mesotrophic lakes. Ecological Network Analysis indices proved to be suitable tools for evaluating ecosystem properties linked to ecosystem stability (as resistance or resilience) in different trophic status environments. An interesting possibility would be to consider using these indices as ecological status indicators for climate change effect studies.

### Conflict of interest statement

The authors declare that the research was conducted in the absence of any commercial or financial relationships that could be construed as a potential conflict of interest.
